# Metabolomic characteristics and related pathways in patients with different severity of COVID-19: a systematic review and meta-analysis

**DOI:** 10.7189/jogh.15.04056

**Published:** 2025-02-28

**Authors:** Chenghao Bi, Junjie He, Yu Yuan, Shumei Che, Ting Cui, Li Ning, Yubo Li, Zhiying Dou, Liwen Han

**Affiliations:** 1 State Key Laboratory of Component-based Chinese Medicine, Tianjin University of Traditional Chinese Medicine, Tianjin, China; 2 Department of Clinical Laboratory, The Second Hospital of Tianjin Medical University, Tianjin, China; 3 School of Pharmaceutical Sciences & Institute of Materia Medica, Shandong First Medical University & Shandong Academy of Medical Science, Jinan, China

## Abstract

**Background:**

Despite advances in metabolomic research on COVID-19, existing studies have small sample sizes and few have comprehensively described the metabolic characteristics of patients with COVID-19 at each stage. In this systematic review, we aimed to summarise the similarities and differences of biomarkers in patients with COVID-19 of different severity and describe their metabolic characteristics at different stages.

**Methods:**

We retrieved studies from PubMed, Embase, Web of Science, and the Cochrane Library published by October 2022. We performed a meta-analysis on untargeted and targeted metabolomics research data, using the ratio of means as the effect size. We compared changes in metabolite levels between patients with varying severity and controls and investigated sources of heterogeneity through subgroup analyses and meta-regression analysis.

**Results:**

We included 22 cohorts from 21 studies, comprising 2421 participants, including COVID-19 patients of varying severity and healthy controls. We conducted meta-analysis and heterogeneity analysis on the 1058 metabolites included in the study. The results indicated that, compared to the healthy control group, 23 biomarkers were associated with mild cases (*P* < 0.05), 3 biomarkers with moderate cases (*P* < 0.05), and 37 biomarkers with severe cases (*P* < 0.05). Pathway enrichment analysis revealed significant disturbances in amino acid metabolism, aminoacyl-tRNA biosynthesis, primary bile acid biosynthesis, pantothenate and CoA biosynthesis, the tricarboxylic acid cycle, taurine and hypotaurine metabolism, and nitrogen metabolism in patients with mild, moderate, and severe disease. Additionally, we found that each severity stage exhibited unique metabolic patterns (all *P* < 0.05) and that the degree of metabolic dysregulation progressively worsened with increasing disease severity (*P* < 0.05)

**Conclusions:**

The results of our meta-analysis indicate the similarities and differences of biomarkers and metabolic characteristics of patients with different severity in COVID-19, thereby providing new pathways for the study of pathogenesis, the development precise treatment, and the formulation of comprehensive strategies.

**Registration:**

PROSPERO: CRD42022369937.

COVID-19 is a respiratory infectious disease caused by severe acute respiratory syndrome coronavirus 2 (SARS-CoV-2) [1,2]. According to World Health Organization (WHO) data, as of 15 February 2023, more than 750 million people worldwide have been diagnosed with COVID-19 and more than 6.8 million people have died, highlighting its impact on human health and society [3]. Affected individuals can be either asymptomatic or can demonstrate various clinical symptoms. In the latter case, the patients can be classified by disease severity as mild patients, moderate patients, severe patients, and critical patients [4]. Although over 80% of COVID-19 patients experience mild symptoms, studies have found that the condition can rapidly progress from mild to severe, especially in the absence of adequate medical care [5]. However, there is limited understanding of the physiological changes associated with COVID-19 under different symptom conditions.

Metabolism refers to the entirety of chemical reactions within an organism, and metabolites are the chemical entities transformed during cellular metabolic processes which serve as a direct reflection of biochemical activity and are highly sensitive to disturbances in the body caused by the onset and progression of diseases [6]. Research has indicated that viruses rely entirely on the host cell's energy and metabolic resources to drive the various stages of viral infection [7].

As a relatively new field of research, metabolomics provides a faster and more accurate method for the study and diagnosis of infectious diseases. By qualitatively and quantitatively analysing the changes in the activities of small molecule metabolites (such as glucose, amino acids, and fatty acids) and their metabolic pathways, researchers can effectively analyse the metabolic regulation and modification rules of specific biological events [8]. In the context of SARS-CoV-2, studies have suggested that the virus induces characteristic molecular changes which, when investigated in patients with different severity of symptoms, may contribute to our understanding of COVID-19 and the development of more effective therapies [9].

The discovery of metabolites increased dramatically with the emergence of metabolomic studies of biological samples from patients with COVID-19 [10]. This led to confusion about biomarkers in COVID-19, such as pyridoxal (PL) which showed trends of up-regulation in patients with mild disease and continuous down-regulations in those with moderate and severe symptoms (*i.e.* with increasing disease severity) [11]. Such unclear biomarkers may hinder a deep understanding of the pathophysiological processes of diseases and could also lead to an inability to accurately distinguish between different stages or severity levels of a disease, thereby affecting diagnostic precision [12].

This left a need for a comprehensive investigation of the biological significance of such substances in patients of different severity levels. In clinical research, biomarkers have demonstrated differing trends, with some showing only weak associations with COVID-19, possibly resulting in the identified biomarkers having limited diagnostic significance or even being erroneous. This may be due to heterogeneity in study design, lack of adequate follow-up intervals, or the use of different biomarker analysis platforms for different studies.

Therefore, we wanted to conduct a meta-analysis of clinical untargeted and targeted metabolomics studies on COVID-19 based on existing research, as well as systematically review and perform a meta-analysis of current findings. Our goal was to identify candidate differential biomarkers with high stability, strong reproducibility, and significant changes within the existing COVID-19 metabolic profiles, which can be applied for precision treatment, exploration of disease mechanisms, and development of therapeutic drugs.

## METHODS

We reported our findings per the PRISMA statement [13] and registered our protocol in PROSPERO (CRD42022369937). We did not seek ethical approval, for our study, as we only used data from published research.

### Sources and search strategy

We searched PubMed, Embase, Web of Science, and the Cochrane Libary for records published by October 2022. We used a search strategy comprising keywords such as ‘COVID-19’ and ‘metabonomics’ or ‘metabolomics’ (Table S1 in the **Online Supplementary Document**). Two authors (CHB and JJH) independently screened their titles/abstracts, followed by their full texts for eligibility and discussed discrepancies with a third author (YY). They also screened the reference lists of all relevant records for records that may have been overlooked in the previous stages.

### Study selection

To be included, studies had to have adopted a metabolomics-based approach in patients with COVID-19 (cohort studies, case-cohort studies, case-control or clinical trials); used high-throughput metabolomics techniques such as nuclear magnetic resonance, gas chromatography (GC), liquid chromatography (LC), mass spectrometry (MS) or a combination thereof to identify metabolites in biological samples; provided means (x̄) and standard deviations (SDs) of biomarker concentrations or sufficient data so they could be calculated directly; included patients with different severity of COVID-19; written English. For articles reporting data on the same cohort or sample, we included those with the most complete data.

We excluded basic experimental studies on infants and children, pregnant women, and animal or cell lines; studies without access to valid data; and conference abstracts, case reports, case series, letters, editorials, subject guides, academic articles, systematic reviews or literature overviews, brief communications, or technical notes.

### Data extraction

The two authors (CHB, JJH) independently extracted the following data from the eligible studies into an extraction sheet: authors; year of publication; type of study design; region; subject characteristics (including sample size and group comparisons, sample type); experimental methods; metabolomics data, including those for the control, mild, moderate, and severe disease groups. They discussed discrepancies with a third researcher (YY) whenever they occurred.

### Risk assessment

Three investigators independently assessed the risk of bias for case-control studies using the Newcastle-Ottawa scale (NOS) [14]. Before the formal scoring, they studied the user guide for the tool and developed a standardised procedure to ensure consistency in the interpretation and application of each scoring item. Two researchers (CHB, JJH) independently assessed the studies, cross-checking their results and discussing any inconsistencies between themselves or (if necessary) with a third researcher (YY).

The NOS rates a study based on its selection of the study population, comparability between groups, and exposure factors. Studies that meet the requirements can receive up to nine points; we thus assessed and scored the quality of the studies independently on a nine‐point scale Points were summed, and studies with scores <3 were considered to be of low quality.

### Statistics and analysis

We conducted reproducibility analysis on COVID-19-related biomarkers of different severity levels and visualised the results using an Upset plot. Biomarkers that appear more than three times and conform to the data format were included in the subsequent analysis, the results of which were then presented in forest plots. We performed the meta-analysis of biomarkers using RevMan, version 5.4.1 (Cochrane Collaboration, Oxford, UK). We performed pathway enrichment analysis and correlation analysis on the biomarkers using the Kyoto Encyclopedia of Genes and Genomes and Pearson analysis, respectively.

A meta-analysis comparing mean difference (MD), standardised mean difference (SMD), and ratio of means (RoM) indicated that, compared to traditional meta-analysis methods using MD and SMD as summary measures for continuous outcomes, the results obtained using the RoM were similar, with no significant difference in heterogeneity [15]. Moreover, using RoM as a continuous outcome effect size can avoid some clinical limitations associated with the MD method, such as the inability to handle results expressed in different units [15]. We thus used the RoM as a substitution effect size for meta-analysis and normalised the effect size to log(ROM).

We pooled the effect sizes using an inverse variance random effects model with statistical significance set at *P* < 0.05 and a fixed effects model. We assessed the heterogeneity of the included studies using the *P*-value of Cochran’s Q and the *I^2^* statistic [16], defining significant heterogeneity as a *P* ≤ 0.10 and *I^2^*>50%, respectively, and low heterogeneity as *P* > 0.10 and an *I^2^<*50%. To further explore potential sources of heterogeneity, we performed subgroup analysis of different groups of biomarkers (frequency of occurrence >4) based on different sample types (plasma, serum, urine) and metabolomics analysis modalities (targeted/untargeted). Next, we performed meta-regression analysis on biomarkers based on sample size to investigate whether there is a linear association between the variables/covariates and the combined effect sizes.

## RESULTS

### Research inclusion process

**Figure 1:**
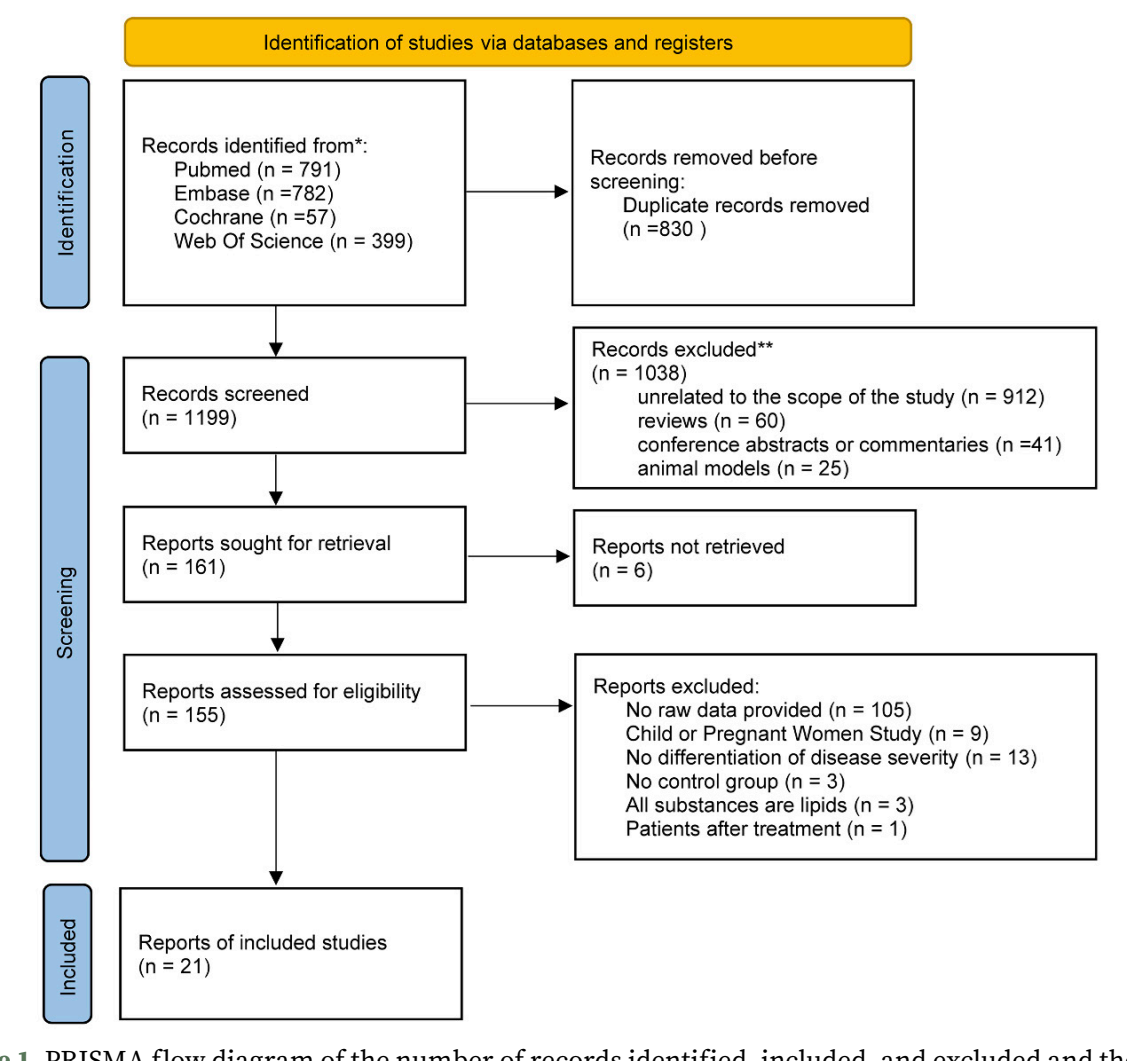
PRISMA flow diagram of the number of records identified, included, and excluded and the reasons for exclusions.

We retrieved 2029 records from PubMed, Embase, Web of Science, and Cochrane. After deduplication, two authors (CHB, JJH) screened the titles and abstracts of the remaining 1199 records, leaving 161 for full-text assessment. Of these, 105 did not have raw data, nine were associated with children or pregnant women, 13 did not classify the degree of disease, three studies did not have a control group, three identified substances that were all lipids, one intervened with subjects, and six did not have an accessible full text. Finally, we included 21 records comprising 22 cohorts in this systematic evaluation (**Figure 1**). All studies used case-control methods.

### Characteristics of included studies

The 22 cohorts involved three types of biological samples, with 15 using plasma samples, five using serum samples, and two using urine samples. Fifteen cohorts used LC-MS, two used nuclear magnetic resonance, two used GC-MS, one used the CE-MS platform, and one combined GC and LC-MS. One cohort used a combined GC and CE-MS, 14 used an untargeted metabolomics approach, and eight cohorts used a targeted metabolomics approach. The studies were published between 2020 and 2022 and comprised 2421 participants. Of these, 853 were controls, 559 had mild symptoms, 536 were moderate disease patients and 473 were severe diseases patients (**Table 1**).

**Table 1:** Basic characteristics of included studies

Study, year, reference	Country	Control group	Mild group	Moderate group	Severe group	Sample type	Method
Herrera-Van Oostdam et al., 2021 [17]	Mexico	37	41	NA	NA	Plasma	Targeted metabolomics
Xue et al., 2022 [18]	China	17	40	NA	8	Plasma	Targeted metabolomics
Xiao et al., 2021 [19]	China	17	14	NA	23	Serum	Targeted metabolomics
Albóniga et al., 2022 [20]	Spain	6	11	11	5	Plasma	Untargeted metabolomics
Byeon et al., 2022 [21]	USA	182	183	NA	139	Plasma	Untargeted metabolomics
Krishnan et al., 2021 [22]	Sweden	21	29	12	NA	Plasma	Untargeted metabolomics
Bi et al., 2022 (urine) [23]	China	27	NA	44	20	Urine	Untargeted metabolomics
Bi et al., 2022 (serum) [23]	China	24	NA	30	6	Serum	Untargeted metabolomics
López-Hernández et al., 2021 [24]	Mexico	39	40	42	40	Plasma	Targeted metabolomics
Chen et al., 2020 [25]	China	12	42	NA	20	Plasma	Targeted metabolomics
Su et al., 2020 [26]	USA	133	45	52	24	Plasma	Untargeted metabolomics
Ambikan et al., 2022 [27]	Sweden	21	NA	29	12	Plasma	Untargeted metabolomics
Jing et al., 2022 [28]	China	102	NA	161	27	Urine	Untargeted metabolomics
Song et al., 2020 [29]	China	26	18	19	13	Plasma	Untargeted metabolomics
Ceballos et al.,2022 [30]	Spain	15	NA	64	47	Plasma	Untargeted metabolomics
Correia et al., 2022 [31]	Brazil	57	21	22	10	Plasma	Targeted metabolomics
Shen et al., 2020 [9]	China	25	NA	NA	21	Serum	Untargeted metabolomics
Wu et al., 2020 [5]	China	10	14	NA	11	Plasma	Untargeted metabolomics
Barberis et al., 2020 [32]	Italy	26	NA	NA	19	Plasma	Untargeted metabolomics
Danlos et al., 2021 [33]	France	27	23	21	NA	Plasma	Targeted metabolomics
Jia et al., 2022 [34]	China	20	18	13	12	Serum	Untargeted metabolomics
Caterino et al., 2021 [35]	Italy	9	20	16	16	Seum	Targeted metabolomics

NA – not applicable

### Quality assessment

Our quality assessment showed a low risk of bias (score ≥4) for all case-control cohorts (n = 22). In general, the identification of controls and comparability between groups were clearly explained in all cohorts, while the inclusion and exclusion criteria of diseases were defined in all but one (**Table 2**).

**Table 2:** Quality assessment by the NOS for all shortlisted case-control studies (n = 22)

	Selection				Comparability		Exposure			
**Study, year, reference**	**Is the case definition adequate?**	**Representativeness of the cases**	**Selection of controls**	**Definition of controls**	**Comparability of cases and controls on the basis of the design or analysis**		**Ascertainment of exposure**	**Same method of ascertainment for cases and controls**	**Non-response rate**	**Scores**
Herrera-Van Oostdam et al. 2021 [17]	Y	Y	Y	Y	Y	Y	Y	Y	Y	9
Ambikan et al., 2022 [27]	Y			Y	Y	Y	Y	Y		6
Correia et al., 2022 [31]	Y			Y	Y	Y	Y	Y	Y	7
Shen et al., 2020 [9]	Y		Y	Y	Y	Y	Y	Y	Y	8
Wu et al., 2020 [5]	Y	Y		Y	Y	Y	Y	Y	Y	8
Barberis et al., 2020 [32]	Y		Y	Y	Y	Y	Y	Y	Y	8
Danlos et al., 2021 [33]	Y	Y	Y	Y	Y	Y	Y	Y		8
Jia et al., 2022 [34]	Y	Y		Y	Y	Y	Y	Y	Y	8
Caterino et al., 2021 [35]	Y		Y	Y	Y	Y	Y	Y		7
Xue et al., 2022 [18]	Y	Y	Y	Y	Y	Y	Y	Y		8
Xiao et al., 2021 [19]	Y	Y	Y	Y	Y	Y	Y	Y		8
Albóniga et al., 2022 [20]	Y		Y	Y	Y	Y	Y	Y	Y	8
Byeon et al., 2022 [21]	Y	Y	Y	Y	Y	Y	Y	Y	Y	9
Krishnan et al., 2021 [22]	Y	Y	Y	Y	Y	Y	Y	Y	Y	9
Bi et al., 2022 (urine) [23]	Y	Y	Y	Y	Y	Y	Y	Y		8
Bi et al., 2022 (serum) [23]	Y	Y	Y	Y	Y	Y	Y	Y		8
López-Hernández et al., 2021 [24]	Y	Y	Y	Y	Y	Y	Y	Y	Y	9
Chen et al., 2020 [25]	Y	Y		Y	Y	Y	Y	Y	Y	8
Su et al., 2020 [26]				Y	Y	Y		Y		4
Jing et al., 2022 [28]	Y		Y	Y	Y	Y	Y	Y		7
Song et al., 2020 [29]	Y	Y	Y	Y	Y	Y	Y	Y	Y	9
Ceballos et al., 2022 [30]	Y	Y		Y	Y	Y	Y	Y	Y	9

### Biomarkers reproducibility analysis

**Figure 2:**
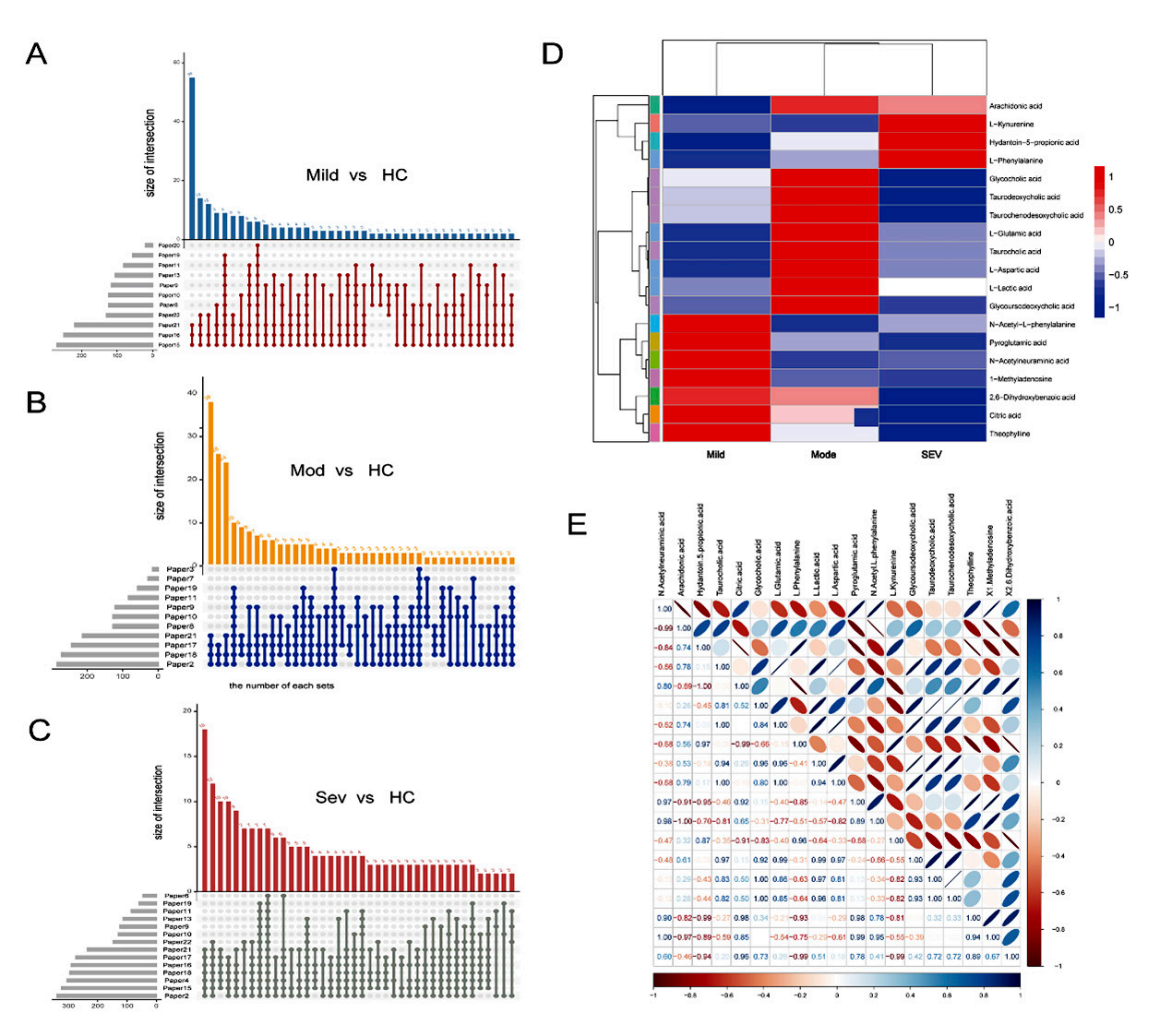
Biomarkers analysis. **Panels A–C.** Reproducibility analysis of metabolites in different groups. **Panel D.** Heat map analysis of three groups of shared biomarkers. **Panel E.** Correlation analysis of three groups of shared biomarkers.

We did not consider biomarkers with poor reproducibility (*i.e.* those appearing in fewer than three studies in reproducibility analysis), which ensured the reliability and consistency of the analysis results, thereby avoiding potential biases or erroneous conclusions due to insufficient data reproducibility. By focussing on biomarkers with higher reproducibility, we were able to more confidently assess their potential roles and clinical significance in the disease. After analysis, we identified 317 mild biomarkers, 332 moderate biomarkers, and 409 severe biomarkers (**Figure 2**, Panels A–C).

The results indicate differences in biomarkers among COVID-19 cohorts of varying severity levels. For example, citrate levels were significantly upregulated in patients with mild COVID-19, but as the disease progresses to moderate and severe stages, the expression of citrate markedly decreased (**Figure 2**, Panel D). This pattern suggests a potential association between citrate levels and the progression of COVID-19, indicating that changes in citrate metabolism may play a role in the disease's severity. We also found that the correlation coefficient between citric acid and lactic acid reached −0.99, suggesting that significant metabolic abnormalities in the tricarboxylic acid (TCA) cycle may have occurred during COVID-19 infection (**Figure 2**, Panel E).

### Meta-analysis

To explore the correlation of biomarkers with different severity of COVID-19, we performed a meta-analysis and heterogeneity analysis of reproducible biomarkers (frequency ≥3). We analysed 317 candidate biomarkers associated with mild cases, of which 24 were significantly different and less heterogeneous, with combined statistical significance (*P* < 0.05 for *Z*; *P* > 0.05 for χ^2^; *I^2^*<50%), We therefore identified them as mild biomarkers. Of the 332 candidate biomarkers associated with mild cases, we identified 21 as moderate disease severity biomarkers (moderate disease biomarkers), with combined statistical significance (*P* < 0.05 for *Z*; *P* > 0.05 for χ^2^; *I^2^*<50%). Of 409 biomarkers associated with severe cases, we identified 37 severe disease biomarkers with combined statistical significance (*P* < 0.05 for *Z*; *P* > 0.05 for χ^2^; *I^2^*<50%).

### Subgroup analysis

To explore the findings of the main meta-analysis, we conducted a subgroup analysis to determine whether the biological sample type and metabonomic analysis method contribute to the heterogeneity of the research results. More specifically, we performed subgroup analysis based on different biospecimen types (plasma, serum, urine) for the above-mentioned biomarkers (frequency of occurrence >4) which had significant differences and high heterogeneity (*P* < 0.05 for *Z*; *P* > 0.05 for χ^2^; *I^2^*<50%). The results of the subgroup analysis indicate that the differences in effect sizes among the biomarker groups were statistically significant (*P* ≤ 0.05). The combined effect of each sub-combination interacted with the types of biological samples, suggesting that different metabolomics analysis methods contributed to the heterogeneity.

Subsequently, we performed subgroup analysis based on different metabolomics analysis approaches (targeted/untargeted) for biomarkers (frequency of occurrence >4) that had shown significant differences and high heterogeneity (*P* < 0.05 for *Z*; *P* > 0.05 for χ^2^; *I^2^*<50%). There was a significant statistical difference in the amount of effect after the combination of those biomarkers, suggesting an interaction between subgroups and effect sizes with biological sample types and consequently indicating that different metabolomic analyses contributed to the heterogeneity of results among studies.

### Meta-regression analysis

Subsequently, for biomarkers with significant differences but substantial heterogeneity (number of studies ≥10), we conducted a meta-regression analysis based on sample size (*P* < 0.05 for *Z*; *P* > 0.05 for χ^2^; *I^2^*<50%). This analysis aimed to explore the relationship between sample size, inter-study heterogeneity, and the overall effect size. We found that only two of the 49 biomarkers were significant (*P* ≤ 0.05), indicating an association between sample size and between-study heterogeneity. A positive regression coefficient suggests a positive correlation between sample size and outcome effects (lactic acid: coef = 0.0048126 > 0; L-methionine: coef = 0.0054491 > 0). We found no statistical significance for the remaining metabolites, suggesting that metabolite-related heterogeneity and outcome variables may not have a corresponding relationship with sample size.

### Pathway enrichment analysis

**Figure 3:**
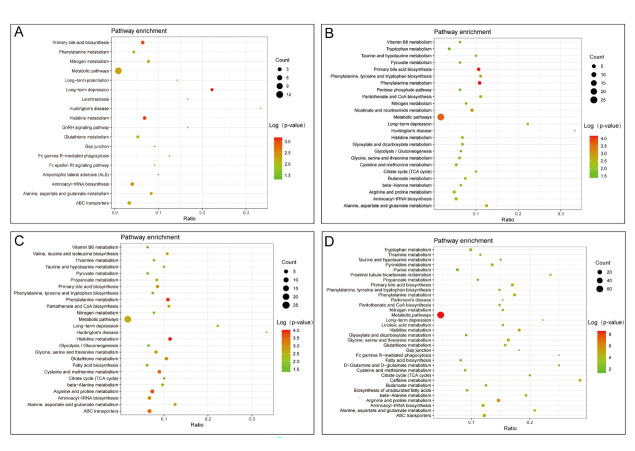
Enrichment analysis of metabolites pathway with COVID-19. **Panel A.** Pathway enrichment of shared biomarkers. **Panel B.** Pathway enrichment of mild disease biomarkers. **Panel C.** Pathway enrichment of moderate disease biomarkers. **Panel D.** Pathway enrichment of severe disease biomarkers.

To characterise the abnormal metabolic pathways in patients with different severity of COVID-19, we performed Kyoto Encyclopedia of Genes and Genomes enrichment analysis to identify metabolic pathways that play an important role in different groups (**Figure 3**). The results showed that amino acid metabolism (arginine and proline metabolism, histidine metabolism, phenylalanine metabolism, glycine, serine and threonine metabolism, β-alanine metabolism, alanine, aspartate and glutamate metabolism, tryptophan metabolism, phenylalanine, tyrosine, and tryptophan biosynthesis, cysteine and methionine metabolism), aminoacyl-tRNA biosynthesis, primary bile acid biosynthesis, pantothenate and CoA biosynthesis, TCA cycle, taurine and hypotaurine metabolism, and nitrogen metabolism were significantly disrupted in all disease groups. However, nicotinate and nicotinamide metabolism, as well as pentose phosphate pathway, were only found to be altered in patients with mild disease. Valine, leucine, and isoleucine biosynthetic pathways were altered only in patients with moderate disease. In those with severe forms of COVID-19, the metabolic disorders were more severe, while the metabolic pathways that were altered were D-glutamine and D-glutamate metabolism, gap junction, linoleic acid metabolism, biosynthesis of unsaturated fatty acids, and gap junction.

## DISCUSSION

In this review, we included 22 cohorts from 21 studies and conducted a meta-analysis of 1,058 metabolites. The analysis involved 2421 participants, including 853 healthy controls, 559 mild cases, 536 moderate cases, and 473 severe cases of COVID-19. We sought to identify the similarities and differences in biomarkers and their metabolic profiles among patients with varying severity levels of COVID-19.

### The role of metabolic disorder in the treatment of COVID-19

Our data did not show significant changes between mild and moderate individuals, indicating that SARS-CoV-2 infections causing mild symptoms do not significantly affect the serum metabolome and related metabolic pathways in these patients. In contrast, the differences were much more pronounced in severe cases.

The TCA cycle provides metabolites for proteins, lipids, and nucleotides, as well as metabolites that regulate the post-translational modifications of control group proteins and other proteins [36]. Our analysis suggests that changes in the systemic flux of mitochondrial TCA cycle intermediates may stem from metabolic pathway dysregulation in COVID-19 patients, potentially related to mammalian target of rapamycin/hypoxia-inducible factor-1 signalling and glycolytic regulation caused by mitochondrial dysfunction [37], making it a candidate for dysregulation by COVID-19 [38].

Amino acids are essential signalling molecules that regulate energy and metabolic homeostasis [39]. A large body of research has confirmed that amino acid metabolism dysregulation is a key feature in the onset and progression of COVID-19, as well as a pathogenic factor that can lead to inflammation, oxidative stress, immune response deficiencies, mitochondrial abnormalities, and other complications [40,41]. In COVID-19 patients, the energy mechanism of mitochondrial adenosine triphosphate (ATP) production appears to be partially suppressed, suggesting that SARS-CoV-2 infection induces a shift in metabolism from aerobic respiration to lactate fermentation [42]. It has further been reported that viral infections, including SARS-CoV-2, can enhance glycolytic flux and increase the conversion of pyruvate to lactate [42]. Viruses can target glycolysis by regulating the expression of glucose transporters, which is crucial for the activation of immune cells during the host cell response [43]. In addition, the metabolism and concentrations of sugars and amino acids (such as glucose, mannose, glutamine, and glutamate) play a crucial role in cellular metabolic homeostasis and are also targeted during viral replication [44]. Glutamine catabolism is considered a carbon source for other human DNA and RNA viruses. Researchers have proposed that metabolic reprogramming of the amino acid glutamine in SARS-CoV-2 may trigger pathogenic mechanisms. Therefore, improving mitochondrial dysfunction and amino acid metabolism dysregulation in COVID-19 patients may become a potential therapeutic approach for treating COVID-19.

The meta-analysis showed that n-acetylneuraminic acid, arachidonic acid, phenylalanine, lactic acid, and kynurenine changed significantly in all COVID-19 groups compared to healthy controls, and showed a consistent up-regulation trend with disease severity (RoM>1). N-acetylneuraminate is the predominant form of sialic acid in mammals. Previous studies have found that serum sialic acid is associated with cardiovascular mortality [45,46]. It triggers myocardial injury both in vitro and in vivo by activating the Rho-ROCK signalling pathway through binding to RhoA and Cdc42 [47]. Chu and colleagues [48] found that coronaviruses can utilise sialic acid to attach to and enter human lung epithelial cells. Additionally, sialic acid-mediated cross-reactivity with host immune collection also plays a role in the immune response during different pathological stages of coronavirus infection [49]. Our meta-analysis shows that n-acetylneuraminate shows a continuous increase with the severity of the disease, suggesting that as the severity of the condition worsens, the incidence of cardiovascular diseases may significantly rise. This is an important factor to consider in patient prognosis.

Arachidonic acid can form anti-inflammatory mediators, such as anti-inflammatory lipoxin A4 (LXA4), during acute inflammation and infection, and such substances play a crucial role in regulating viral replication and altering host innate and adaptive immune responses [50]. It has been shown that LXA4 and arachidonic acid can regulate SARS-CoV-2 infection by inhibiting viral entry, suppressing viral replication, down-regulating angiotensin-converting enzyme 2 expression and suppressing pro-inflammatory cytokines [51]. It has been reported that SARS-CoV-2 invasion in mild patients only triggers a specific mild immune response [52], while immune suppression has been observed in the early stages of COVID-19 disease [53]. A previous study found that the synthesis of arachidonic acid was relatively enhanced under the overall decrease in fatty acids [54]. This discovery emphasises that the arachidonic acid pathway is the central regulator of inflammatory response [55]. Compared to the activated immune response observed in patients with severe COVID-19, those with milder forms of the disease have a milder immune response, which may help them overcome the potentially life-threatening cytokine storm caused by systemic inflammatory overreaction [56]. Therefore, the levels of arachidonic acid in this group may not significantly increase, nor trigger severe cytokine storms or tissue damage. In patients with moderate COVID-19, however, the metabolites of arachidonic acid may show a marked increase, especially in cases of cytokine storm and excessive activation of inflammatory responses. Studies have shown that derivatives of arachidonic acid, such as prostaglandin E2 and leukotriene A4, may induce immune cells (*e.g.* macrophages, T-cells) to secrete large amounts of inflammatory cytokines (such as interleukin-6 or tumor necrosis factor-α), thereby exacerbating the inflammatory response and tissue damage. In severe COVID-19 patients, this excessive immune response often manifests as systemic inflammatory response and organ dysfunction. Our meta-analysis suggests that arachidonic acid could serve as a potential diagnostic biomarker for different severities of COVID-19.

In addition, inflammatory cytokines promote muscle decomposition and release phenylalanine for gluconeogenesis during COVID-19 infection to supply energy demand during infection. Luporini and colleagues’ research proves that phenylalanine is positively related to the severity of COVID-19 and suggests that it is a marker of disease severity [57]. Lactate is a typical biomarker of mitochondrial metabolic dysfunction [58]. In COVID-19 patients, the energy mechanism of mitochondrial ATP production appears to be partially inhibited, suggesting that SARS-CoV-2 infection induces a shift in metabolism from aerobic respiration to lactate fermentation [59]. In severe COVID-19, intense inflammatory responses are associated with tissue hypoxia, leading to the release of high levels of lactate from muscle tissue; this may be the reason for the elevated lactate levels as the condition progresses [60,61].

Citric acid, as a key intermediate of the TCA cycle, plays a stabilising role in energy metabolism and cellular function, it serves as a bridge between carbohydrate and fatty acid metabolism, promoting the proliferation and differentiation of immune cells such as B cells [62].In severe cases of COVID-19, increased oxidative stress may affect the normal functioning of the TCA cycle, thereby disrupting the production and utilisation of citrate [63]. In our study, citric acid showed a consistent downregulation with disease severity (RoM<1) in patients with COVID-19, suggesting that our energy supply was disturbed during the SARS-CoV-2 infection. Notably, PL showed an up-regulation trend in the light patient cohort and its continuous down-regulation in the medium and heavy cohorts with increasing disease severity. Under the condition of a specific kinase (pyridoxal kinase enzyme), PL can be converted to active PL phosphate [64]. Some studies have reported that PL phosphate can alleviate the symptoms of COVID-19 infection; for example, it has been suggested that PL can improve the immune system function by preventing cytokine storms and oxidative stress in the early stage of infection [65]. Therefore, we speculate that with the aggravation of the disease, the inflammatory reaction of moderate and severe COVID-19 patients accelerates the consumption of PL and PL phosphate.

We further analysed the biomarkers that appeared individually in different severity levels. Significant changes in 1-methylnicotinamide and methionine were uniquely found in mild COVID-19 patients compared to controls. 1-methylnicotinamide, an endogenous substance with anti-inflammatory and anti-thrombotic characteristics, may alleviate the persistent symptoms of fatigue in patients with COVID-19 by improving skeletal muscle energy metabolism [66,67]. Methionine has been reported to potentially regulate SARS-CoV-2 assembly by a mechanism that interferes with RNA polymerase [68], which could provide a potential target for antiviral therapy in mild patients. L-isoleucine, 5-hydroxylysine, s-adenosylhomocysteine, and spermine were found to be significant only in the moderate severity group. The elevated level of S-adenosylhomocysteine can be regarded as a marker of the risk of lung injury in COVID-19 patients, and it is likely to be a factor related to the development of inflammatory process and the reduction of glutathione, the main cellular antioxidant [69].

Thirty-seven unique metabolites (including a variety of amino acids, glucose, and bile acid products) were significantly altered in severe COVID-19 patients. We speculate that this may be due to the higher number of complications in severe patients, where the host's response to infection is driven by multi-system dysfunction, leading to greater metabolic disturbances [25].

Down-regulation of tryptophan may trigger inflammation, an important risk factor for morbidity and mortality, as it leads to defects in components of the innate and adaptive immune system, resulting in a decrease in the immune response with age and an increase in the severity of infections [70]. The results of our meta-analysis showed that, in severe COVID-19 patients, kynurenine was up-regulated and tryptophan was down-regulated, indicating that the ratio of canine uric acid to tryptophan was increased. Notably, studies have suggested that the increase in the ratio of kynurenine/tryptophan is highly correlated with the severity of COVID-19 [71,72].

Several studies have found that taurine plays a critical role in regulating immune system health and exerting antioxidant effects, which is attributed to its anti-inflammatory properties by inhibiting cytokine release [73,74]. The results of our meta-analysis showed a significant decrease in taurine in severe COVID-19 patients, suggesting that more taurine was depleted as the disease worsened.

In addition, we found a significant down-regulation of sphingosine-1-phosphate (S1P) observed in severe COVID-19 patients, which is consistent with the results of previous clinical studies [75,76]. S1P is a signalling molecule that exerts multiple actions through its specific G protein-coupled receptor. It is thought to be important for the protection of vascular integrity, as well as the disrupted endothelial barrier in the lung during COVID-19 [77]. Thus, compounds with high selectivity to specific S1P receptors or capable of interfering with the phosphorylation step of S1P by sphingosine may be a potential therapeutic approach. Research has indicated that dysregulated levels of N-acetyl ornithine metabolism, which may be related to the mechanism of SARS-CoV-2 infection, and dysregulation of the ornithine cycle are significantly associated with inflammation and coagulation in severe COVID-19 patients, which may be a potential mechanism for COVID-19 pathogenicity [78].

### The influence of heterogeneous sources on metabolomics

Metabolomics primarily involves the study of endogenous metabolites (with a molecular weight of <1500 Da) in samples such as tissues, blood, and urine, in order to obtain metabolic profile information related to health and its changes [79]. The main processes of metabolomics include experimental design, biological sample preparation, data collection, and data analysis. Each stage can be influenced by various factors such as the subject's ethnicity, age, gender, weight, diet, medication, sample storage and handling, and analytical methods. These controllable and uncontrollable factors can introduce variability and affect the interpretation of metabolic data [80], as they can all be potential sources of heterogeneity.

With this in mind, we conducted heterogeneity analysis with the aim of providing some reference for the standardisation of metabolomics procedures. The results indicated that different biological sample types may be sources of this metabolic heterogeneity. Both urine and blood can reflect real-time changes in the body; however, there were certain differences in the metabolites detected across different sample types (plasma, serum, urine) – for example, we found that glutamate decreased in serum, but increased in urine. It is worth noting here, however, that content and significance of metabolites vary across different sample types [22], while inconsistent sample collection, storage, and pre-processing can also lead to experimental biases.

### Strengths, limitation, and future research directions

However, we should note some limitations as well. Metabolomics research is divided into untargeted metabolomics and targeted metabolomics. Different analytical techniques have their advantages and disadvantages in terms of metabolite categories, coverage, and sensitivity [81]. Additionally, chemical contaminants and signal redundancy may result in raw data that cannot be matched with databases, leading to difficulties in accurate metabolite identification and quantification [82].

We further speculate that other reasons affect the results of our analysis. Racial, lifestyle, and clinical factors may all have an impact on metabolism in patients with COVID-19 [83–85]. Because specific data on individual patient clinical characteristics were not presented or measured in the included observational studies, we could not adjust for them as potential confounders. Moreover, the studies included in our analysis did not specify whether they excluded patients with comorbidities, so we were also unable to explain the differences caused by patient comorbidities. Further, different sample pre-processing methods for different biological sample types might have led to differences in the results of metabolite detection. In addition, the initial data from the included studies were transformed and harmonised using the appropriate formulae to incorporate more data. Inevitably, however, these conversion methods suffer from a certain estimation bias, which in turn introduces heterogeneity.

Furthermore, due to limited number of included studies, we were unable to conduct stratified analysis to evaluate differences in specific geographic regions or populations. In addition, despite our strict inclusion and exclusion criteria, we observed heterogeneity in our results due to differences in biological sample types, control types, data conversion, and metabolomics methodology, as well as differences in unknown confounding factors. Therefore, the impact of these factors needs to be considered in future studies related to COVID-19 biomarkers.

We believe that the following measures can be taken to improve the consistency and reliability of metabolomics research:

1. Method standardisation: adopt widely recognized, standardised metabolomics analysis protocols (*e.g.* methods recommended by the Human Metabolome Database or mass spectrometry imaging frameworks)
2. Cross-platform calibration: when using different instruments (*e.g.* LC-MS and GC-MS), perform cross-platform calibration of results through standards and calibration samples to improve data consistency.
3. Design prospective studies: systematically assess the impact of methodological variations on results by designing well-controlled, prospective studies. These strategies can help minimize variability and enhance the reproducibility of metabolomics findings.

We believe that, through the standardisation of samples and methods, the repeatability of metabolomics research can be significantly improved. This will not only reduce the heterogeneity between studies, but also lay a foundation for the establishment of widely applicable biomarkers.

## CONCLUSIONS

This systematic review and meta-analysis explored the similarities and differences of biomarkers and metabolic characteristics of patients with mild, moderate, and severe COVID-19, *i.e.* different levels of COVID-19 severity. N-acetylneuraminic acid, arachidonic acid, L-phenylalanine, L-kynurenine, and Citric acid were significantly changed in all groups and showed a continuous trend of up-regulation or down-regulation with disease severity. The reason for the different trends of PL in different cohorts of patients with COVID-19 may be inextricably linked to its co-modulatory role in the inflammatory response pathway. In addition, significant changes in 1-methyl nicotinamide, 2-amino-3-hydroxy propanoic acid, and l-methionine were found uniquely in the mild patients compared to the healthy control group. L-isoleucine, 5-hydroxylysine, s-adenosylhomocysteine, and spermine were significantly changed only in patients with moderate disease. For those with severe COVID-19, 37 unique metabolites were significantly altered. The main reason could be that these patients often have complications, and the host's response to infection leads to multiple system dysfunction, which leads to high metabolic disturbance. The results of the pathway enrichment analysis showed that the dominant metabolic pathways of COVID-19 differed among different patient groups. The results of the subgroup and meta-regression analyses indicate that the type of biological sample, the metabolomics analysis method, and the number of cohort sample characteristics are all major sources of heterogeneity. In all, the results of our meta-analysis clarified the similarities and differences of biomarkers and metabolic characteristics of patients with different severity of COVID-19, providing new ground for the study of the pathogenesis of neo-coronary and the precise treatment of patients.

## Additional material


Online Supplementary Document
