# Supplementary material for: Metabolomic characteristics and related pathways in patients with different severity of COVID-19: a systematic review and meta-analysis
Source: J Glob Health. 2025 Feb 28;15:04056. doi: 10.7189/jogh.15.04056 (PMC11869518; doi:10.7189/jogh.15.04056)
Supplement: Online Supplementary Document [file jogh-15-04056-s001.pdf]

Supplement to: Bi C, He J, Yuan Y, Che S, Cui T, Ning L, Li Y, Dou Z, Han L. Metabolomic characteristics and related pathways in patients with different severity of COVID-19: a systematic review and meta-analysis. J Glob Health. 2025;15:04056.

Table S1: Literature search strategy of the study.

Table S2. Sub-group analysis of different factors (types of biological samples, metabolomics analysis methods).

Table S3. Meta-regression analyses of metabolites, comparing Mild-patients with COVID-19 and controls.

Table S4. Meta-regression analyses of metabolites, comparing Mod-patients with COVID-19 and controls.

Table S5. Meta-regression analyses of metabolites, comparing Sev-patients with COVID-19 and controls.

Table S1: Literature search strategy of the study.

	NO.	Terms
		COVID-19
Pubmed	#1	((COVID-19[MeSH Terms]) OR (COVID 19) OR (SARS-CoV-2 Infection) OR (Infection AND SARS-CoV-2) OR (SARS CoV 2 Infection)OR (SARS-CoV-2 Infections) OR (2019 Novel Coronavirus Disease) OR (2019 Novel Coronavirus Infection) OR (2019-nCoV Disease) OR (2019 nCoV Disease) OR (2019-nCoV Diseases) OR(Disease AND 2019-nCoV) OR (COVID-19 Virus Infection) OR (COVID 19 Virus Infection) OR (COVID-19 Virus Infections) OR (Infection AND COVID-19 Virus) OR (Virus Infection AND COVID-19) OR (Coronavirus Disease 2019) OR (Disease 2019 AND Coronavirus) OR (Coronavirus Disease-19) OR (Coronavirus Disease 19) OR (Severe Acute Respiratory Syndrome Coronavirus 2 Infection) OR (SARS Coronavirus 2 Infection) OR (COVID-19 Virus Disease) OR (COVID 19 Virus Disease) OR (COVID-19 Virus Diseases) OR (Disease AND COVID-19 Virus) OR (Virus DiseaseAND COVID-19) OR (2019-nCoV Infection) OR (2019 nCoV Infection) OR (2019-nCoV Infections) OR(Infection AND 2019-nCoV) OR (COVID19) OR (COVID-19 Pandemic) OR (COVID-19 Pandemic) OR (Pandemic AND COVID-19) OR (COVID-19 Pandemics))
		Metabolomics/ Lipidomics
	#2	(("Metabolomics"[MeSH Terms] OR "Metabolome"[MeSH Terms] OR "Lipidomics"[MeSH Terms] OR "metabolo*"[All Fields] OR "metabonom*"[All Fields] OR "metabolic profile*"[All Fields] OR ("metabolic"[All Fields] AND "profile*"[All Fields]) OR "lipidom*"[All Fields]))
	#3	#1 AND #2
		Metabolomics/ Lipidomics
	#1	'metabolomics'/exp OR 'metabolome'/exp OR 'lipidomics'/exp OR 'metabolo*' OR 'metabonom*' OR 'metabolite profile*' OR ('metabolite' AND 'profile*') OR 'lipidom*'
		COVID-19
Embase	#2	'coronavirus disease 2019'/exp OR 'covid 19' OR 'sars cov 2 infection' OR ('infection' AND 'sars-cov-2') OR (sars AND cov AND 2 infection) OR (covid AND 19) OR 'sars cov 2 infections' OR ('covid 19 virus' AND infection) OR (2019 AND novel AND coronavirus AND disease) OR (2019 AND novel AND coronavirus AND infection) OR (2019 AND ncov disease) OR '2019 ncov disease' OR '2019 ncov diseases' OR ('disease' AND '2019-ncov') OR (covid AND 19 virus AND infection) OR ('covid 19 virus' AND infections) OR ('infection' AND 'covid-19 virus') OR ('virus infection' AND 'covid-19') OR ('disease 2019' AND 'coronavirus') OR (coronavirus AND 'disease 19') OR (coronavirus AND disease AND 19) OR (severe AND acute AND respiratory AND syndrome AND coronavirus AND 2 infection) OR (sars AND coronavirus AND 2 AND infection) OR ('covid 19 virus' AND disease) OR (covid AND 19 virus AND disease) OR ('covid 19 virus' AND diseases) OR ('disease' AND 'covid-19 virus') OR ('virus disease' AND 'covid-19') OR '2019 ncov infection' OR (2019 AND ncov infection) OR '2019 ncov infections' OR ('infection' AND '2019-ncov') OR covid19 OR 'covid 19 pandemic' OR (covid AND 19 pandemic) OR ('pandemic' AND 'covid-19') OR (coronavirus AND disease AND 2019)
	#3	#1 AND #2
Cochrane	#1	***Metabolomics/ Lipidomics***

		MeSH descriptor: [Metabolomics] explode all trees OR MeSH descriptor: [Metabolome] explode all trees OR MeSH descriptor: [Lipidomics] explode all trees OR “Metabolomics” OR “Metabolomic” OR “Metabonomics” OR “Metabonomic” OR “Metabolome” OR “Metabolomes” OR “Metabolic Profile” OR “Metabolic Profiles” OR (“Profile” AND “Metabolic”) OR (“Profiles” AND “Metabolic”) OR “Lipidomics” OR “Lipidomic” OR “Lipidome” OR “Lipidomes”
		COVID-19
	#2	MeSH descriptor: [COVID-19] explode all trees OR(COVID-19)OR(COVID 19)OR(SARS-CoV-2 Infection)OR(SARS CoV 2 Infection)OR(SARS-CoV-2 Infections)OR(2019 Novel Coronavirus Disease)OR (2019 Novel Coronavirus Infection)OR (2019 nCoV Disease)OR (COVID-19 Virus Infection)OR (COVID 19 Virus Infection)OR(COVID-19 Virus Infections)OR (Coronavirus Disease 2019)OR (Coronavirus Disease-19)OR (Coronavirus Disease 19)OR(Severe Acute Respiratory Syndrome Coronavirus 2 Infection)OR (SARS Coronavirus 2 Infection)OR(COVID-19 Virus Disease)OR (COVID 19 Virus Disease)OR(COVID-19 Virus Diseases)OR(2019 nCoV Infection)OR (COVID19)OR(COVID-19 Pandemic)OR(COVID 19 Pandemic)OR(COVID-19 Pandemics)OR(Infection AND SARS-CoV-2)OR(Virus Infection AND COVID-19)OR(Disease 2019 AND Coronavirus)OR(Disease AND COVID-19 Virus)OR(Virus Disease AND COVID-19)OR(Pandemic AND COVID-19)
	#3	#1 AND #2
Web of Science		***Metabolomics/ Lipidomics***
	#1	(TS=("Metabolomics") OR TS=("Metabolome") OR TS=("Lipidomics") OR ALL=("metabolo*") OR ALL=("metabonom*") OR ALL=("metabolite profile*") OR (ALL=("metabolite") AND ALL=("profile*")) OR ALL=("lipidom*"))
		COVID-19
	#2	(TS=(COVID-19) OR ALL=(COVID 19) OR ALL=(SARS-CoV-2 Infection) OR ALL=("Infection"AND "SARS-CoV-2") OR ALL=(SARS CoV 2 Infection) OR ALL=(SARS-CoV-2 Infections) OR ALL=(2019 Novel Coronavirus Disease) OR ALL=(2019 Novel Coronavirus Infection) OR ALL=(2019-nCoV Disease) OR ALL=(2019 nCoV Disease) OR ALL=(2019-nCoV Diseases) OR ALL=("Disease"AND" 2019-nCoV") OR ALL=(COVID-19 Virus Infection) OR ALL=(COVID 19 Virus Infection) OR ALL=(COVID-19 Virus Infections) OR ALL=("Infection"AND"COVID-19 Virus") OR ALL=("Virus Infection"AND"COVID-19") OR ALL=(Coronavirus Disease 2019) OR ALL=("Disease 2019"AND"Coronavirus") OR ALL=(Coronavirus Disease-19) OR ALL=(Coronavirus Disease 19) OR ALL=(Severe Acute Respiratory Syndrome Coronavirus 2 Infection) OR ALL=(SARS Coronavirus 2 Infection) OR ALL=(COVID-19 Virus Disease) OR ALL=(COVID 19 Virus Disease) OR ALL=(COVID-19 Virus Diseases) OR ALL=("Disease"AND"COVID-19 Virus") OR ALL=("Virus Disease"AND" COVID-19") OR ALL=(2019 nCoV Infection) OR ALL=(2019-nCoV Infections) OR ALL=(COVID19) OR ALL=(COVID-19 Pandemic) OR ALL=(COVID 19 Pandemic) OR ALL=("Pandemic"AND"COVID-19")
	#3	#1 AND #2

Table S2. Sub-group analysis of different factors (types of biological samples, metabolomics analysis methods).

Search	Sample type	Search	Metabonomics analysis mode
Herrera-Van Oostdam AS, 2021	plasma	Herrera-Van Oostdam AS, 2021	Targeted Metabolomics
Ambikan AT, 2022	plasma	Correia BSB, 2022	Targeted Metabolomics
Correia BSB, 2022	plasma	Danlos FX, 2021	Targeted Metabolomics
Wu D, 2020	plasma	Xue M, 2022	Targeted Metabolomics
Barberis E, 2020	plasma	López-Hernández Y, 2021	Targeted Metabolomics
Danlos FX, 2021	plasma	Chen YM, 2020	Targeted Metabolomics
Xue M, 2022	plasma	Caterino M, 2021	Targeted Metabolomics
Albóniga OE, 2022	plasma	Xiao N, 2021	Targeted Metabolomics
Byeon SK, 2022	plasma	Bi X, 2022（urine）	Untargeted Metabolomics
Krishnan S, 2021	plasma	Jing Y, 2022	Untargeted Metabolomics
López-Hernández Y, 2021	plasma	Ambikan AT, 2022	Untargeted Metabolomics
Chen YM, 2020	plasma	Wu D, 2020	Untargeted Metabolomics
Su Y, 2020	plasma	Barberis E, 2020	Untargeted Metabolomics
Song JW, 2020	plasma	Albóniga OE, 2022	Untargeted Metabolomics
Ceballos FC，2022	plasma	Byeon SK, 2022	Untargeted Metabolomics
Shen B, 2020	serum	Krishnan S, 2021	Untargeted Metabolomics
Jia H, 2022	serum	Su Y, 2020	Untargeted Metabolomics
Caterino M, 2021	serum	Song JW, 2020	Untargeted Metabolomics
Xiao N, 2021	serum	Ceballos FC，2022	Untargeted Metabolomics
Bi X, 2022（serum）	serum	Shen B, 2020	Untargeted Metabolomics
Bi X, 2022（urine）	urine	Jia H, 2022	Untargeted Metabolomics
Jing Y, 2022	urine	Bi X, 2022（serum）	Untargeted Metabolomics

Table S3. Meta-regression analyses of metabolites, comparing Mild-patients with COVID-19 and controls.

Mild-Variables	Number of obs	coef.[95% CI]	*p*-Value[a]	Adj R-squared
HMDB0000094	11	0 .00058[0.0002603,0.0014203]	0.1530	10.53%
HMDB0000148	11	-0.0075026[-0.0363471,0.0213419]	0.1140	-7.45%
HMDB0000159	13	0.0001511[-0.0013548,0.001657]	0.8290	-11.31%
HMDB0000190	11	0.0048126[-0.0039539,0.0073443]	0.0020	64.89%
HMDB0000684	10	0.0033423[-0.004,0.0106384]	0.3220	0.66%
HMDB0000696	10	0.0054491[0.0031359,0.0077622]	0.0010	77.73%

Table S4. Meta-regression analyses of metabolites, comparing Mod-patients with COVID-19 and controls.

Mod-Variables	Number of obs	coef.[95% CI]	*p*-Value[a]	Adj R-squared
HMDB0000094	11	-0.0001543[-0.0057028,0.0053942]	0.9510	-13.53%
HMDB0000148	10	-0.108834[-0.3688108,0.1511428]	0.3630	-0.78%
HMDB0000158	10	0.0006894[-0.0031802,0.0045589]	0.6920	-18.90%
HMDB0000159	11	-0.0019576[-0.0110319,0.0071166]	0.6370	-8.78%
HMDB0000190	11	-0.0055713[-0.021139,0.0099963]	0.4390	-3.87%
HMDB0000243	11	0.0002222[-0.010034,0.0104785]	0.9620	-31.05%

Table S5. Meta-regression analyses of metabolites, comparing Sev-patients with COVID-19 and controls.

Sev-Variables	Number of obs	coef.[95% CI]	*p*-Value[a]	Adj R-squared
HMDB0000064	15	-0.0019976[-0.0095122,0.0055171]	0.5760	-5.91%
HMDB0000079	10	0.0005679[-0.0010676,0.0022033]	0.4460	-57.07%
HMDB0000094	17	0.0003062[-0.0016766,0.0022891]	0.7470	-11.97%
HMDB0000122	13	0.0052072[-0.0603125,0.0707269]	0.8640	-8.90%
HMDB0000123	15	-0.0003504[-0.0049185,0.0042177]	0.8710	-28.72%
HMDB0000127	10	0.0021991[-0.0067782,0.0111764]	0.5880	-9.07%
HMDB0000138	11	-0.0012014[-0.0248473,0.0224444]	0.9110	-12.25%
HMDB0000148	15	-0.0209329[-0.0966097,0.054744]	0.5600	-4.86%
HMDB0000158	15	-0.0000259[-0.0034027,0.0033509]	0.9870	-12.35%
HMDB0000159	17	-0.0005205[-0.0033209,0.0022798]	0.6980	-6.97%
HMDB0000177	16	0.0008946[-0.0008437,0.0026329]	0.2880	-0.72%
HMDB0000190	15	-0.0020204[-0.0091289,0.005088]	0.5500	-4.92%
HMDB0000191	12	-0.0006035[-0.005987,0.0047801]	0.8080	-10.01%
HMDB0000192	11	0.0021298[-0.0058609,0.0101205]	0.5610	-8.81%
HMDB0000195	11	-0.0132695[-0.1110532,0.0845143]	0.7660	-10.48%
HMDB0000197	12	-0.000456[-0.0019505,0.0010384]	0.5120	0.00%
HMDB0000208	11	-0.0011594[-0.006982,0.0046633]	0.6630	-9.68%
HMDB0000251	13	0.0008173[-0.0017568,0.0033914]	0.4990	-13.05%
HMDB0000254	14	-0.0014067[-0.0063154,0.0035021]	0.5440	-7.07%
HMDB0000259	10	-0.0004724[-0.0030687,0.0021238]	0.6860	-26.70%
HMDB0000267	12	0.0008189[-0.0028504,0.0044882]	0.6300	-17.09%
HMDB0000289	12	0.0006503[-0.0021195,0.00342]	0.6120	-9.39%

HMDB0000292	11	-0.0021473[-0.0098065,0.0055119]	0.5420	-6.64%
HMDB0000296	11	0.000572[-0.0025087,0.0036526]	0.6840	-11.52%
HMDB0000619	10	-0.000361[-0.0049119,0.0041899]	0.8590	-24.73%
HMDB0000625	11	-0.0220689[-0.112097,0.0679592]	0.5930	-7.48%
HMDB0000641	13	0.0003987[-0.0016039,0.0024013]	0.6700	-12.21%
HMDB0000684	13	0.0074128[-0.024301,0.0391266]	0.6170	-6.80%
HMDB0000708	11	-0.0029591[-0.0182519,0.0123338]	0.6720	-9.40%
HMDB0000714	11	-0.0008813[-0.0128997,0.0111371]	0.8720	-18.39%
HMDB0000725	10	0.0011845[-0.0017951,0.0041641]	0.3860	-2.36%
HMDB0000766	10	0.0000813[-0.0043496,0.0045122]	0.9670	-15.59%
HMDB0000767	10	0.0005639[-0.0013565,0.0024842]	0.5170	-10.12%
HMDB0000904	13	0.0006565[-0.002844,0.0041571]	0.6880	-14.26%
HMDB0000929	16	0.000949[-0.0016108,0.0035089]	0.4400	-5.77%
HMDB0002829	10	-0.0006426[-0.0068825,0.0055973]	0.8180	-17.75%
HMDB0003331	11	-0.0017876[-0.0089058,0.0053307]	0.5840	-8.03%
